# Force production during the sustained phase of Rugby scrums: a systematic literature review

**DOI:** 10.1186/s13102-020-00174-z

**Published:** 2020-05-25

**Authors:** Eric Martin, George Beckham

**Affiliations:** Kinesiology Department, California State University Monterey Bay, 100 Campus Center, Seaside, California 93933 USA

**Keywords:** Rugby union, Scrummaging, Biomechanics

## Abstract

**Background:**

Since World Rugby changed the laws regarding scrums in the 2013–2014 season, the sustained push phase of the scrum has increased in tactical importance. Therefore, the purpose of this systematic literature review was to examine the biomechanical demands during the sustained push phase of individual, unit, and full pack scrummaging.

**Methods:**

Pubmed, EBSCO (specifically and simultaneously searching Academic Search Premier, CINAHL, and SPORTDiscus), and Google Scholar were searched for any research that presented force production in a live or simulated rugby scrum. Study quality was appraised using the National Institute of Health’s Quality Assessment Tool for Observational Cohort and Cross-Sectional Studies. Recorded scrum forces, positioning of players including joint angles, and testing procedures were extracted and narratively synthesized.

**Results:**

Twenty six studies were included in the review. 50% of included studies were rated good, 31% fair, and 19% poor. Major limitations included not reporting any effect size, statistical power, or reliability. Reported group mean values for average sustained forces against a machine generally ranged from 1000 to 2000 N in individual scrums and 4000–8000 N for full packs of male rugby players older than high school age. Individuals seem to optimize their force generation when their shoulders are set against scrum machine pads at approximately 40% of body height, with feet parallel, and with knee and hip angles around 120°. A 10% difference in pack force seems to be necessary for one pack to drive another back in the scrum, but little data exist to quantify differences in force production between winning and losing packs during live scrums. Data collection within studies was not standardized, making comparisons difficult. There is a lack of data in live scrums, and the current research indicates that machine scrums may not replicate many of the demands of live scrums. There is a lack of data for female rugby players.

**Conclusions:**

This review indicates an optimal individual body position for players to strive to achieve during scrummaging, consisting of a low body height (40% of stature) and large extended hip and knee angles (120° each).

## Background

In rugby (including union, league, and 7 s), when play is restarted after a dead-ball infringement such as a knock-on or other stoppage, the two teams contest for the ball with a scrum [1, 2]. For example, in the fifteen-a-side variant of rugby union (the setting in which most research has been conducted), eight players from each team bind together to form a pack, which then opposes the other team’s pack, giving each team an opportunity to gain possession of the ball. During the scrum, each pack attempts to push forward with more force than the other team to gain ball possession and territory and to disrupt the other team from successfully handling the ball. While winning many scrums does not necessarily mean a team will win the game [3], a successful scrum (whether the team is putting the ball in or manages to steal the put in from the other team) can provide a strong platform for scoring tries [4]. Thus, the ability to apply greater force against the ground and against the opposing pack during the scrum may be of great interest to players and coaches, in order for them to gain a tactical advantage in the game.

Under the old laws of rugby union (prior to the 2013–14 season), opposing front rows started further apart, resulting in greater peak impact forces upon engagement. A pack that could generate greater peak impact forces than the opposing pack during the engagement phase (often defined as the moment from initial contact until 1 s after peak force occurs [5, 6]) had a tactical advantage. However, high impact forces were related to injury rates in the scrums [7], especially the catastrophic injuries [8]. Thus, over the years, World Rugby has changed the rugby union laws about scrummaging with the specific goal of decreasing the impact forces and thus decreasing injury rates. One law modification tried was a staggered scrum engagement, in which the opposing front rows engaged each other before the rest of the pack bound on. While this resulted in lower impact force, it also created greater scrum instability and therefore still an unsafe scrum [9, 10]. To better understand the risk of injury and effect of potential scrum rule modifications pertaining to the process of pack engagement, World Rugby commissioned a series of studies [5, 6, 11]. The findings from these studies led World Rugby to adopt the “crouch, bind, set” method of engaging a scrum, which reduces the peak forces on engagement while still providing good scrum stability [5, 11].

Due to the law changes about scrum engagement, the engagement phase of the scrum has not only become safer but of less tactical importance. The sustained push phase of the scrum (from the moment the ball is put in until the scrum has ended), which has received less research attention, has now become of greater tactical importance. Furthermore, the rule changes regarding the scrum have resulted in scrums lasting longer, from an average of 7.5 s prior to the law change to 10.8 s by the 2016 season [12]. Additionally, since this law change, there has been a significant increase in the number of scrums performed during English Premiership and Six Nations competitions [12, 13]. Thus, an examination of biomechanical demands specifically during the sustained push phase of the scrum is warranted. The purpose of the current systematic review was to describe the forces created by individuals, forward units (e.g. the tight five), and full packs during the sustained push phase of the scrum.

## Methods

**Fig. 1:**
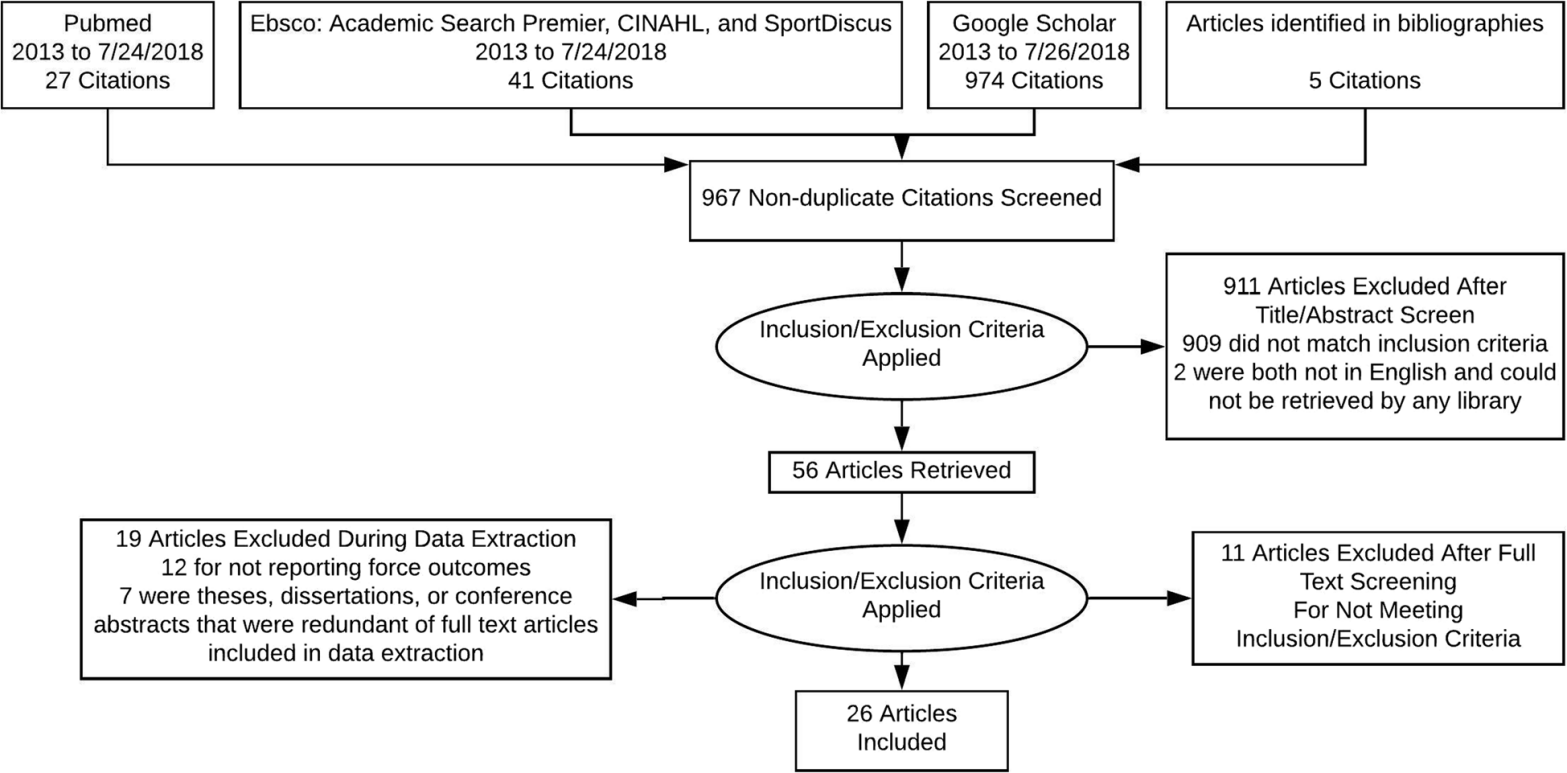
Search Flow Diagram

This systematic review was conducted in line with the PRISMA guidelines. The databases searched were Pubmed, EBSCO (simultaneously searching Academic Search Premier, CINAHL, and SPORTDiscus), and Google Scholar. The most recent literature review on scrum biomechanics, which focussed on injuries, completed their search in 2013 [7]. Therefore, the current search queried databases on research published after January 2013. The search term set RUGBY AND SCRUM was used in all searches. All results returned were copied into a spreadsheet. The spreadsheet included which database the citation originated from, the title of every article returned, and a hyperlink to each article. Entries were sorted by article title and duplicates removed. All titles and abstracts were screened for inclusion based on the criteria of interest, which were that the article report force production by one or more individuals in a rugby scrum position, whether live or simulated against a machine. Journal articles, theses, dissertations, and conference abstracts were all included. Whenever the content of a thesis or dissertation was found to duplicate corresponding journal articles (e.g. common in doctoral thesis-by-publication), the thesis or dissertation was excluded in favor of the journal article. Similarly, conference abstracts were excluded if they duplicated the content of a published journal article. The full text articles of all remaining studies were retrieved and screened to ensure they reported force produced during a scrum. Bibliographies of both review articles identified at any time during the search and the full text articles retrieved were read to identify any further articles that may need to be included. During this process, articles published prior to January 2013 were retrieved. The final flow of articles included can be seen in Fig. 1.

Data that were extracted included type of research product; study design; specific code of rugby; country where research took place; playing level of subjects; time of season during which data were collected; sample size, sex, age, playing position(s), rugby experience and resistance training experience of players; equipment used to capture force production; surface tested on; positioning and set up of players including joint angles; testing procedures; and outcomes of force measurement. In cross-sectional studies that tested forces under different engagement conditions, the data from the condition that most closely resembled the modern procedure (the referee calls crouch-bind-set, and the props must bind on the opposite team’s prop at the call of bind and maintain their bind) was extracted for reporting. If studies reported longitudinal changes in scrummaging force data across a season, only baseline force outcomes were extracted. Prior research has consistently demonstrated that force production in the scrum is strongly related to the player’s body mass [9, 14–16] and that as playing level increases, player body mass increases [17, 18]. Therefore, to be able to make comparisons between playing levels independent of the increased mass of players at higher levels, when possible, we normalized force production by dividing the reported average force produced by the sample by the average reported body mass of the players or forward pack performing the test. When necessary, all reported mean values were converted to Newtons by multiplying kilograms by 9.81.

The National Institute of Health’s (NIH’s) Quality Assessment Tool for Observational Cohort and Cross-Sectional Studies was used to rate study quality. Question number four, which asked if subjects were recruited from the same or similar populations, was not used as the scope of the review (limited to only rugby players in the scrum) made this question automatically yes for all included studies. Question number six, which asked if outcome exposures were measured prior to the outcome being measured, was deemed not necessary because all potential exposures were categorical and did not require scalar measurement (for example, if a player was in high school or professional). While all questions were considered when making a final judgement, the following questions were focussed on: #1: Clear research question; #2: Clearly defined study population; #5: Sample or effect size reported; #9: Validity and reliability of independent variables reported; #11: Validity and reliability of dependent variables reported; and #14: Accounted for key confounders. Both authors independently used the tool to rate each study, then compared their results. For any discrepancies in initial ratings, studies were re-reviewed by both authors together and a consensus agreed upon based on a strict interpretation of the NIH’s guidelines.

## Results

### Study quality

Study quality was generally high, as indicated by the scores from the NIH’s Quality Assessment Tool for Observational Cohort and Cross-Sectional Studies (Table 1); 13 studies were rated good, eight rated fair, and five rated poor. The main limitation amongst studies, which is common in sports research, was sample size. Especially in high-level athletes, researchers are often limited to samples of convenience, i.e. the one local team they have access to. To make up for this inherent limitation, the NIH recommends reporting effect sizes, confidence intervals, or some indicator of statistical power. Only 11 of the studies reviewed included some determination of statistical power, primarily using effect size; those which did not include any indicator of statistical power limited the veracity of their results. Another limitation of the current literature overall is the sparse amount of reliability testing done; only one study of a homogeneous group of international level French players has examined the inter-trial reliability of testing scrum force production [29]. In pilot testing prior to the main study, Lacome asked eight players to perform three individual maximal isometric scrums against a scrum machine; the scrums lasted 5 s, and 6 min of rest were given between repetitions. From this pilot testing, Lacome determined that maximal force could be reproduced under these conditions with an ICC = 0.8. Related to reproducibility and validity, some studies did not have subjects perform multiple scrum repetitions during testing to at least account for potential invalid trials, which may reduce the reliability of their results.

**Table 1:** Quality ratings of studies

Study	Q1. Clear research question	Q2. Clear population	Q3. ≥50% participation of eligible population	Q5. Sample or effect size	Q7. Sufficient time frame	Q8. Examined levels of outcome exposure	Q9. Validity and reliability of independent variables	Q10. Exposures assessed over time	Q11. Dependent variables clear, valid, and reliable	Q12. Assessors blind	Q13. ≤20% Follow up loss	Q14. Adjusted for key confounders	Overall quality rating
Babault et al., 2007 [19]	Y	Y	CD	N	Y	N	Y	Y	Y	NR	NR	N	Fair
Bayne & Kat, 2018 [20]	Y	Y	CD	Y	Y	NA	Y	Y	Y	NR	NR	Y	Good
Birch, 2016 [21]	Y	N	CD	Y	Y	NA	Y	Y	Y	NR	NR	Y	Fair
Cazzola et al., 2015 [5]	Y	Y	CD	Y	Y	Y	Y	Y	Y	NR	NR	Y	Good
Clayton, 2016 [22]	Y	Y	CD	N	Y	N	Y	Y	Y	NR	NR	Y	Good
Cochrane et al., 2017 [23]	Y	Y	CD	N	Y	Y	Y	Y	Y	NR	NR	Y	Good
Dobbs, 2017 [24]	Y	Y	CD	N	N	NA	NA	Y	N	NR	NR	N	Poor
Du Toit et al., 2004 [14]	N	Y	CD	N	N	NA	Y	Y	Y	NR	NA	N	Fair
Du Toit et al., 2005 [9]	N	Y	CD	N	Y	Y	Y	Y	N	NR	NR	N	Poor
Ferrandino et al., 2015 [25]	Y	N	CD	N	Y	Y	Y	Y	Y	NR	N	N	poor
Green et al., 2016 [26]	Y	Y	CD	N	N	Y	Y	N	Y	N	NR	Y	Good
Green, Kerr, Dafkin, et al., 2017 [15]	Y	Y	CD	Y	Y	Y	Y	N	N	NR	NR	Y	Fair
Green, Kerr, Olivier, et al., 2017 [27]	Y	Y	CD	Y	Y	Y	Y	N	N	NR	NR	Y	Fair
Green, Dafkin, et al., 2017 [28]	Y	Y	CD	N	Y	Y	Y	N	N	NR	NA	Y	Fair
Hodge, 1981 [10]	N	N	CD	N	Y	Y	N	Y	CD	NR	NR	CD	Poor
Lacome, 2013 [29]	Y	Y	CD	N	Y	Y	Y	Y	Y	NR	NR	Y	Good
Mensaert et al., 2015 [30]	N	Y	CD	N	N	Y	Y	Y	Y	NR	NA	Y	Fair
Milburn, 1990 [31]	Y	Y	CD	N	Y	Y	Y	NA	Y	NR	NR	N	Fair
Morel et al., 2015 [32]	Y	Y	CD	Y	Y	Y	Y	Y	Y	NR	NR	Y	Good
Morel & Hautier, 2017 [33]	Y	Y	CD	Y	Y	Y	Y	Y	Y	NR	NR	Y	Good
Preatoni et al., 2013 [17]	Y	Y	CD	Y	N	Y	Y	Y	Y	NR	NR	Y	Good
Preatoni et al., 2015 [6]	Y	Y	CD	Y	Y	Y	Y	N	Y	NR	NR	Y	Good
Preatoni et al., 2016 [11]	Y	Y	N	Y	Y	Y	Y	Y	Y	NR	NR	Y	Good
Quarrie & Wilson, 2000 [16]	Y	Y	Y	Y	Y	NA	Y	Y	Y	NR	N	Y	Good
Saletti et al., 2013 [34]	Y	N	CD	N	NA	NA	Y	Y	N	NR	NR	CD	Poor
Wu et al., 2007 [35]	Y	Y	CD	N	Y	Y	Y	N	Y	NR	NR	Y	Good

*CD* cannot determine, *NR* Not reported, *NA* not applicable, *Y* yes, *N* no

### Demographic characteristics of samples

After applying inclusion and exclusion criteria, 26 research outputs were included. Of these, 20 were journal articles, four were theses or dissertations, and two were conference abstracts. Twenty five studies were conducted with rugby union players, and one did not specify the code of rugby. The majority were acute experiments (*n* = 11) or observational (*n* = 10), while four were cross-sectional comparison studies, and one was an intervention training study. Most studies sampled South African players (*n* = 6), with British (*n* = 5) and French (*n* = 5) teams also frequently sampled; three studies were conducted in New Zealand, two in Australia, and one study each came from the United States of America, Sweden, Canada, and Taiwan. Several studies were either cross-sectional or their sample included players from multiple playing levels without differentiation; thus, four studies sampled from international level players, 11 from elite/professional players, two from academy and semi-professional teams, 11 from amateur/community clubs, six from universities, six from high schools, and three from elite women’s teams. While the majority of studies did not specify the sex of their participants, we assumed all samples were male except for samples drawn explicitly from elite women’s teams. Most studies did not specify the time of season when testing occurred. Cohrane et al. [23] stated they conducted testing after the competitive season, Green, Kerr, Olivier, et al. [27] performed testing 4 weeks prior to the start of the inter-varsity tournament, Babault et al. [19] started their intervention 2 weeks after the mid-winter break, and Wu et al. [35] assessed players during the competition preparation phase. Sample sizes in each study ranged from only three players up to a study with 432 players drawn from 54 forward packs (and across five playing levels), though the majority of studies recruited fewer than 30 participants. Participants ranged in mean age from 16.6 to 34 years old, with the majority in their early 20s. Twelve studies used the full pack [5, 6, 9–11, 14, 16, 17, 22, 26, 28, 31], while four studies used the front row [23, 30, 33] and two used the tight five only [15, 20]. Two studies recruited a mix of forwards and backs [24, 35]. Six studies did not specify the participants’ playing position [19, 21, 25, 27, 32, 34]. Few studies quantified rugby playing experience of their subjects: Dobbs [24] had an inclusion criteria for university athletes to have played at least 6 months. Green, Dafkin, et al. [28] studied amateur players with an average of 11 years of playing experience, and Clayton [22] sampled players with an average of 8.4 years. The national players Wu et al. [35] studied had played for 9.1 years on average. No study reported the resistance training experience of their sample.

### Equipment used for testing

Most of the studies (*n* = 21) had players scrum against a scrum machine [6, 10, 14–17, 19–23, 25–31, 33–35]. Often the scrum machine was commercially purchased and then instrumented by the researchers, though some researchers built their own apparatus completely from scratch. Three studies used pressure pads or pressure sensor arrays affixed to players’ shoulders to capture forces during live scrums against an opposing pack [5, 9, 11]. One study had subjects push against a safety-squat bar and measured ground reaction forces using a force plate [24], while another study used a fixed yoke [32]. Nine studies had players perform scrums on natural grass turf [5, 9, 11, 15, 20, 26–28, 31], three used synthetic turf [22, 23, 25], two used rubber matting [14, 16], and three were done indoors on lab or track floors [21, 32, 35]. One study had participants stand on a force plate, which the researchers had put skateboard tape on to reduce slippage [24]. Several studies did not report what surface players were tested on. Four studies had players support their feet on sprinter blocks or other wedges [19–21, 32], though most studies just had players in shoes or cleats with no extra foot support. One study, however, had players go barefoot, though this choice was not explained [35].

### Scrum test protocols

Only one study indicated that they provided or required subjects to perform a separate familiarization session [32]. Few studies provided details on the warm up used. Most studies utilized a general self-selected or coach-directed warm up (presumably of dynamic activities like high knees, butt kickers, jogging, etc) [5, 6, 11, 17, 20, 26, 33], and some studies provided athletes with warm up pushes on the scrum machine in addition to or instead of a general dynamic warm up [5, 6, 11, 16, 17, 21, 22, 24–26, 35]. The number of warm-up trials was not typically specified; the few studies that did stated that three to five submaximal trials [22] or eight 50% effort trials [25] were given.

Players were required to push for 5–10s in duration, usually performing two to five scrums per set [16, 19, 21, 22, 26, 30, 32], with a maximum of eights scrums per set [6, 17, 25]. In studies that evaluated scrums under multiple conditions (e.g. varying foot position, before and after a fatigue protocol), multiple rounds of two to five scrums were used [5, 9, 11, 20, 23, 24, 34, 35]. In most cases players rested for 1–2 min between scrum trials [6, 11, 17, 20, 22, 23, 34, 35], though some protocols restricted rest to only 15–24 s [16, 32, 33], and one allowed as long as 4 min between scrums [19]. In studies that tested the scrum under multiple conditions and reported between scrum-set rest periods, the rest between sets of scrums ranged from “at least one minute” to 10 min [5, 6, 11, 20, 23, 35].

### Body positioning in the scrums

The majority of studies allowed players to choose their positioning, though Clayton [22] reported that individuals were set up approximately 0.5 m away from the scrum pad, and Wu et al. [35] controlled the scrummaging height of players and tested them at 36, 38, and 40% of their body height. Most studies did not control or measure the joint angles of participants. Those that did had a wide variety of methodology. Cochrane [23] used a goniometer to set players into knee and hip angles of 120°; Dobbs [24] used a goniometer to set players into knee and hip angles of 90°; Morel [32] used starting blocks to control the position of the player’s feet, and set them far enough away from the yoke so that players pushed with approximate knee and hip angles of 130°. Other studies allowed players to self-select their scrum position, and the researchers measured the resulting joint angles. During the sustained pushing phase of the scrum, Green, Kerr, Dafkin, et al. [15] reported mean hip extension angles ranging from 152.6–155.0°, knee extension angles of 152.4–155°, and ankle angles of 70.5–74.6°, depending on the leg measured. Mensaert [30] measured players at professional, senior, and junior amateur levels during the engagement phase, and found hip extension angles ranging from 143 to 176°, knee extension angles ranging from 97 to 104°, and ankle angles of 90–93°. Quarrie & Wilson [16] found average angles within all forward positions of 123° of hip extension, 107° of knee extension, and 78° at the ankle. With these reported averages, it is worth noting that the standard deviations, i.e. inter-player variability, were sometimes quite high. For example, Green, Kerr, Dafkin, et al. [15] observed standard deviations of 19.6–20.5°, 17–23.9°, and 19.8–20.4° for the hip, knee, and ankle, respectively, while Mensaert and colleagues [30] found the range of hip extension angles to vary by 63–89°, depending on the playing level. The variation in joint angles both between studies and within studies highlights the difficulty in determining a so-called “ideal scrum position”, particularly when considering the large variety of body dimensions both across and within specific rugby playing levels.

Wu and colleagues [35] tested players in multiple positions (making shoulder contact at 40, 38%, or 36% of body height), and found that professional rugby union players produced significantly more force at 40% of body height (*p* < 0.01). However, it was interesting to note that there were no significant differences between the three scrum heights for hip, knee, or ankle angles. In their further analysis of what joint angles occurred at the highest pushing forces during a parallel stance, they found mean angles of 117.6° at the hip, 100.7° at the knee, and 53.9° at the ankle. An investigation by Green, Kerr, Dafkin, et al. [15] found similar results for both height and joint angles among university level tight five forwards. These players self-selected a position so their average back and pelvic height were at 49.2 and 41.5% of their standing stature, respectively, during the sustained push of the scrum. These positions changed little (less than 1%) across the different phases of the scrum. These players had slightly different joint angles in the left and right lower extremities, indicating they were not in a perfectly parallel stance, though likely were not in a purposefully staggered stance either. During the sustained push phase, mean hip extension ranged from 149.9–155.7°, knee extension ranged from 144.5–148.65°, and ankle angle ranged from 76.9–78.6°, depending on the leg measured. Furthermore, this study examined the correlations between the different examined body position characteristics and force production. They found that lower extremity joint angles did not significantly correlate with force production in any phase of the scrum; however, stance width, pelvic height, and back height did significantly correlate with force production, such that a lower body position and wider stance increased force production. Similarly, Quarrie and Wilson found no significant correlations between ankle, knee, and hip angle and individual scrum force [16]. In contrast, Bayne and Kat [20] found that knee angle had a large correlation and hip angle had a very large correlation with individual scrumming force, though they did not report body or pelvic height.

Aside from body position, the only individual scrum technique variation that has been examined is the use of a parallel or staggered foot position. The few research studies that have tried these two conditions have consistently found that the parallel foot stance allows for greater forward force generation against a scrum machine [20, 35]. However, a staggered stance increases lateral forces, which may be of tactical importance or help with scrum stability [20]. The staggered foot position may also reflect the demands of a shifting scrum, as players reposition their feet. For whole-pack technique, the recent investigations have studied how opposing packs come together, with the goal of finding a technique that reduced peak engagement forces and increased the safety of the scrum. From the examined research, the consensus is that the entire pack should be fully bound onto each other before engaging the other team, as opposed to having the opposing front rows engage and then having the other five players engage (this was termed a staggered scrum engagement) in order to preserve scrum stability [9, 10], and that the modern call of crouch-bind-set, where the props must reach out and maintain a full bind with their opposite prop, is effective at significantly reducing the peak engagement force [11]. Additionally, Hodge [10] mentioned that using a hip-bind technique for the locks to bind to the props may “indicate a safer technique for the prop” (page 36) than a crotch binding technique, though there was no difference in pack force production between the techniques used.

### Force production in the scrum

#### Pack force production in live scrums

**Fig. 2:**
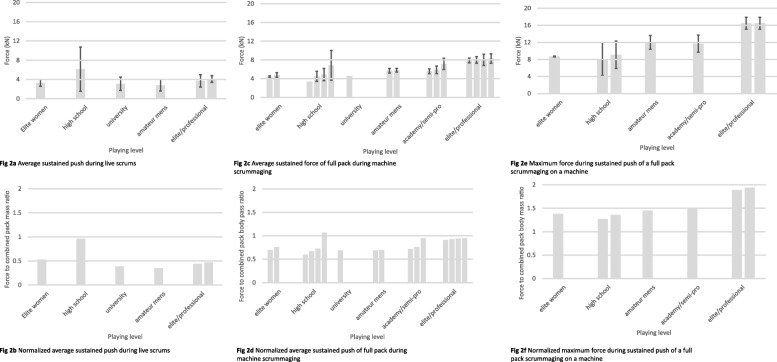
Mean Force Production during Full Pack Scrummaging. Note: Different bars within the same playing level indicate average forces reported from different studies. Error bars indicate standard deviation either from manuscript or, when the manuscript reported standard error of the mean, standard deviation was calculated by the formula $SD= SEM\times \sqrt{n}$. When no error bars are present, that indicates the manuscript gave no indication of error

Reported average forces during live scrums can be seen in Fig. 2a. One surprising result was that there was not an obvious trend for increase in average sustained pack force with increasing playing levels/combined pack weight (Fig. 2a). The one study that tested forces in live scrums of high school players stands out in the graph as an unexpected outlier [9], with those players pushing with over 50% more force than the professionals. We double checked the manuscript to try to determine if there was some error in reporting, but the front row totals were consistent with the reported individual force production. Unlike Preatoni et al. [11] and Cazzola et al. [5], who calibrated their pressure sensors such that they would be optimized for the measurement of scrum forces [36], Du Toit and colleagues [9] failed to explain if a calibration process was used. Additionally, the average combined mass of opposing packs of high school players reported in Du Toi et al. [9] was 1361 kg, compared to an average combined mass of 1771 kg in elite [11] and 1708 kg in professional [5] players, making the finding that the high school players were both absolutely stronger and stronger relative to their mass highly unlikely. Therefore, while we cannot be entirely certain these are or are not plausible values for high school forwards, based on all the other data reported, it seems safe to say that this was an outlier. Ignoring this outlier, normalized average sustained force appears to increase with playing level (elite men were better than amateur and university men; Fig. 2b) as would be expected, given the greater strength [18], lean body mass and overall body mass [37] observed with increasing playing level. We would expect this pattern to exist amongst female rugby players as well, given the high (53%) normalized average sustained force observed in elite female players, but unfortunately no scrum-force data on non-elite female rugby players exist yet to confirm this.

#### Pack force production in machine scrums

While there was not a perfectly clear increase in pack force with increasing playing level in live scrums (Fig. 2a, b), when testing on a scrum machine, there was a clear trend for increases in average sustained pack force with increases in playing level/combined pack weight during machine scrummaging (Fig. 2c). This trend remained when normalizing force by pack weight (Fig. 2d). In a live scrum (Fig. 2a and b), packs generally produce lower forces than they do against a scrum machine (Fig. 2c and d). Assuming that the packs included in each of the studies summarized by Fig. 2a and c are representative of their playing level, it appears that during live scrums, packs only exert about 50% of the force they produced on a scrum machine. While there was no direct correlation analyses between live and machine scrummaging forces, du Toit and colleagues conducted two studies with the same sample of high school players; one study measured full pack pushing forces during live scrums [9] and the other during machine scrummaging [14]. The resultant force during machine scrummaging (i.e. the force applied along the actual angle of push from the players) was 1.79–1.82x higher at max and mean, respectively, compared to the sustained force during live scrummaging.

The most dramatic differences between packs of different playing levels is seen in maximum force produced during the sustained pushing phase of the scrum when scrummaging against a machine (Fig. 2e and f). The large absolute force differences between the men’s elite/professional packs and all other packs may be in large part due to greater mass (Fig. 2e). Overall, there was a trend for greater maximum force and less variability (as indicated by smaller standard deviations) with increases in playing level and pack mass as expected. However, when normalized for pack weight, differences in maximum force during the sustained push phase greatly shrink between all playing levels except elite/professional, who still show a much greater ability to generate force. For example, the ratios of normalized maximum pack force between all comparisons of elite women, high school, amateur, and academy, range from 0.95–1.15, indicating that at these playing levels forwards are able to use their mass to generate force in the scrum to a similar extent. In comparison, the ratios of normalized maximum pack force between professionals and each other playing level range from 1.27–1.46, indicating that the players who make it to the highest level can use their mass much more effectively to generate forces in the scrum. These ratios hint at the likely impact of technique and other factors aside from pack mass on effectively producing pack scrum force. While relative forces can be useful for comparisons between ability level, it is important to note that in competition, it is ultimately the absolute forces produced that will dictate the movement of the scrum. Therefore, regardless of the relative forces produced by a pack, greater absolute forces are of primary importance: a pack able to generate greater absolute forces in the scrum has an advantage over an opposing pack with lower absolute forces even if they produce higher relative forces.

#### Relationship of other variables to scrum force production

In the research so far, total pack mass seems to be the greatest determinant of scrum force production. For example, elite women pushed less than semi-professional men, likely due to the lower mass of the women players rather than them being any lower in skill or body-weight-relative strength; indeed, in live scrummaging the women had a better normalized force production than any other group (except high school, which as previously discussed is likely an anomaly; Fig. 2b). Studies that have correlated combined pack body mass to scrum force production have consistently found that the two factors are highly related [9, 14–16]. Surprisingly, however, scrum force has not been unequivocally correlated with other measures of performance. For example, Green, Kerr, Dafkin, et al. [15] found no significant relationships between vertical jump height (both absolute and normalized to individual’s height and mass) and engagement (r = −.071, *p* = .738), peak (r = .084, *p* = .691) or sustained (r = −.072, *p* = .734) scrum forces among university players. Similarly, among males in the Dunedin premier rugby competition, average sustained force on a scrum machine did not correlate with vertical jump [16]. In contrast, Green, Dafkin, et al. [28] found that the scrum pack with the higher average vertical jump height of individual players in the pack pushed the opposing pack backwards more often. An additional finding in the study by Green, Dafkin, et al. [28] was that combined pack body mass was not a significant determinant of pushing the opposing pack backwards. This was likely due to the similarity in mass between the two packs (difference in the average pack mass was only 4 kg). Furthermore, when looking at the individual scrum trials performed during the study and comparing the combined mass of the two packs, there were occasions when the winning pack weighed 75 kg less than the losing pack, demonstrating that while total body mass may allow for greater force production, greater mass does not guarantee scrum success.

#### Individual player contributions to total pack scrum force

Few studies have examined the contribution of individual players toward full pack force production. Those that have investigated this question have pursued it in two ways: 1) how much force does each unit (front row, second row/locks, back row/flankers and 8-man) contribute during a full pack scrum? And 2) do the forces of individual players scrummaging alone sum to the total force of a full-pack scrum? In answer to the first question, previous research has shown that players do not all contribute equally. Most studies indicate that back row players produce the least force in a full pack scrum, whereas front row players contribute the most force both due to their own force production and transfer of forces from the players behind them [9, 14, 31]. This is partly due to positioning, as flankers only bind on with one shoulder and therefore have less of a platform with which to transmit force straight forward into the pack unit. This disadvantage due to binding position is supported by the finding that in individual scrum trials (single players at a time) against a scrum machine, among amateur men’s players, the back row players did not create significantly less force, either absolute or relative to body mass, when compared to front row or second row players [26]. Du Toit and colleagues found that in both machine and live scrummaging, the front rows produce 40–51% of the average or maximum sustained pack force, locks produced 31–33% of these forces, and back rows contributed 18–27% [9, 14]. These percentages are in line with a review Milburn published in 1993 [38], which indicated that the front rows contributed 42%, the second row 37%, and the flankers 25%. However, there is data to suggest very different contributions of the units within the pack. For example, Milburn [31] tested a high school pack on a scrum machine, starting with only the front row and then adding in players in subsequent trials. The front row produced 3290 N, while the full scrum produced 3370 N, indicating that the front rows alone could produce 98% of the scrum force. Additionally, adding the second rows made a minimal increase in total scrum force (310 N / 8% increase), indicating a minimal rather than nearly equal contribution of the second row to the front row. It was surprising that the 2nd rows did not contribute as much force to the whole pack scrum, as 2nd rows often have as much body mass as front rows and individually have been measured to produce as much if not more force than front rows [14, 16]. However, based on the large swings in horizontal and vertical shear forces with each combination of units, it is possible that this single high school pack had trouble stabilizing with more players and effectively transmitting their forces in a synergistic manner [31]. Due to the much larger sample size in the Du Toit articles [9, 14] (over 200 players), and the agreement with other reviewed research [38], it seems reasonable to conclude that front rows do produce the most scrum force (approximately 45%), with locks contributing approximately 35% and back rows contributing 20%. These data and summaries should be interpreted in light of the samples they represent (mostly high school) and that they were collected 10–40 years ago, and therefore may not represent the modern player, especially at higher playing levels. Equally as important, these contributions need to be examined among female rugby players.

For examining if individual forces sum to total force during a scrum, Quarrie and Wilson [16] first showed that the sum of individual forces do not all get directed through the full pack by testing players on a scrum machine individually and then testing the whole pack on the scrum machine. They found that, on average, only 65% of the force produced by individuals is transferred into a scrum machine when the whole pack scrummed together. This is likely due to loss of force in other directions besides the forward direction (for example, lateral forces created by the angle of the flankers binding onto the props, or shear forces directed in the vertical axis), as well as general instability created by having eight players try to bind together rather than being able to optimally bind against a stationary and fully supportive scrum machine by themselves.Interestingly, while packs exerted 65% of the sum of their individual scrum results on average, there was significant variation between tested scrum packs (range: 52 to 74%). This variation indicates that there is substantial variability, even amongst professional scrum packs, in translating individual scrum performance to group scrum performance. This variability may indicate differences in individual and unit technique. Unfortunately, so far, no research has been conducted to operationally define individual or group technique, nor demonstrate a way to objectively measure it.

#### Force production and scrum success

Only one study compared forces of winning and losing forward packs [28]. Winning was determined as pushing the other pack back about 1.5 m; they did not put in the ball to contest actual possession. Sixteen amateur players performed individual trials against an instrumented scrum machine to test their maximum individual scrum force. Then, players formed into packs using varying combinations of 8 players in their normal position (e.g. the two tight head props always had to tight head prop) and performed live scrums against each other. Green, Dafkin et al. [28] presented their data as the sum of individual scrum forces expressed as percentage of body weight (e.g. if each of the 8 player’s individual scrum force was 200% body weight, then the paper reported the summed value for the entire scrum as 1600% body weight). Green, Dafkin, and colleagues reported that there was an average difference of summed individual scrum force between winning and losing scrums of 182.1% body weight. To make results more comparable with other studies, we calculated absolute forces in Newtons using force data from Table 1 and scrum pack mass data from Table 2 [28]. We divided summed individual forces (in percentage of individual body weight) by 8 to calculate mean individual force (as percentage of body weight) then multiplied by mean pack mass to determine total pack force in kilograms, and finally multiplied by 9.81 to change the units to Newtons. The sum of individual machine scrum trial forces was 19,065 N for winning packs on average and 17,402 N for losing packs on average, for an average difference of 1663 N, or 9%, between winning and losing packs.

Summing individual scrum forces likely does not represent pack force production, because the sum of forces from individuals’ trials is greater than the forces produced during full pack scrummaging [16]. To try to account for this loss of force as well as how much each row in the pack contributes to total scrum force, Green, Dafkin, and colleagues [28] also calculated a pack total force by summing individual scrum force produced on the machine weighted for the percentage contribution expected from their position according to prior research. They performed separate calculations based on research from Du Toit et al. [14] and Milburn [31]. Du Toit [14] indicated that the front row contributes 42%, the second row 37%, and the back row 21% of total scrummaging force while Milburn found that the front row contributes 46%, second row 24%, and back row 30% of total pack force. Thus, Green, Dafkin, et al. [28] weighted player’s individual scrum machine results to create a weighted total. Using these position-specific scaling calculations, Green, Dafkin, et al. [28] found there was a 10–11% average difference in total force between winning and losing packs. Using the absolute force magnitudes we calculated from Green, Dafkin, et al. [28], and adjusting for the magnitude of force loss found by Quarrie and Wilson [16], the forces produced by winning packs in a live scrum are estimated as 1081 N or 9% higher on average (12,392 N for winning packs and 11,311 N for losing packs), closely matching the differences in percentage in row-weighted pack totals between winning and losing scrums [28]. While these magnitudes should be confirmed in future research, all these different methods of calculation indicate a 9–10% difference in force production between packs is associated with driving an opposing pack back 1.5 m, which may provide an advantage in winning possession at the scrum.

#### Individual force production against a scrum machine

**Fig. 3:**
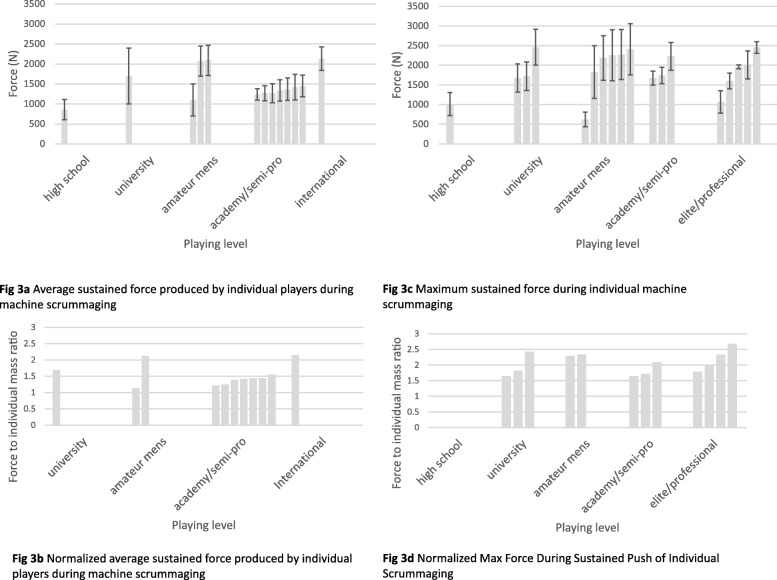
Mean Force Production of Individuals during Machine Scrummaging. Note: Different bars within the same playing level indicate average forces reported from different studies. No study of high school players that presented maximum sustained force provided body mass of samples, therefore normalized max force could not be calculated for this population. Error bars indicate standard deviation either from manuscript or, when the manuscript reported standard error of the mean, standard deviation was calculated by the formula $SD= SEM\times \sqrt{n}$. When no error bars are present, that indicates the manuscript gave no indication of error

When testing players individually against a scrum machine for how much force they could produce, the expected trend of greater force with greater playing level was seen (Fig. 3). However, especially when forces were normalized to individual player body mass, there was a lot of overlap between playing levels, especially for maximum force produced during sustained scrummaging (Fig. 3d).

### Fatigue during repeated scrums

Seven studies were found that examined the effect of fatigue, either from repeated scrums or other activities, on scrum force production. Lacome [29], Morel et al. [32], and Cochrane et al. [23] each examined the effects of repeated scrum trials on scrum force production. Lacome [29] had French National players perform twelve 5 s individual maximal scrums against a machine, with 15 s passive rest between trials (10 s standing, 5 s to get back into position). A statistically significant decrease of 11.7% of average scrum force was seen starting at the 4th scrum, and this lower force production was maintained through the twelfth repetition. Morel et al. [32] asked players competing in the U23 French championships to perform five 5 s maximal isometric individual scrums against a fixed yoke attached to a force sensor, interspersed with 20 s passive rest. In their analysis, they only compared repetitions one, three, and five, finding a significant decrease from trial one to three, and a significant decrease from trial one to five of a 23% reduction. In a follow up to their earlier study, Morel and Hautier [33] tested elite U-23 front row players on individual scrum force in response to fatigue. These players performed six maximal isometric pushes against a BabyScrum ergometer, each lasting 6 s. There were two inter-repetition recovery conditions, and each participant performed both in a two-session randomized crossover design. One condition was passive recovery (standing for 24 s), and the other was active recovery (run for 10s then get ready for the next scrum for 24 s total). In this study, there was not a statistically significant decrease in average force across scrum trials, nor was there a difference between active and passive recovery conditions. Cochrane et al. [23] asked twelve front row players from academy, development, or semi-professional teams to perform three sets of five maximal effort isometric scrums, each lasting 10 s. Players were given 40 s rest between repetitions and 2 min rest between sets. When scrum trials within each set were averaged, there was not a statistical difference between sets one, two and three. However, there was a decrease in scrum force from the first rep to the third rep within set two and a decrease from the first rep to the third and fourth repetitions in set three, indicating growing fatigue across trials. Force data were only presented graphically, and therefore the percentage of loss experienced on the third repetitions of sets two and three can not be accurately estimated.

Other studies examined the effects of various fatigue protocols on scrum performance. Birch [21] examined the fatiguing effects of mental or physical stressors on scrum force. Ten local and/or national rugby union players attended two separate sessions. During each session, they performed a physical and mental fatigue protocol, with the order of protocols randomly determined in a crossover design. They performed one set of five repetitions of isometric maximal scrums before, between, and after the fatigue protocols. The length of rest between repetitions of the scrum test were not specified. The mental fatigue protocol consisted of 30 min of modified incongruent Stroop word tasks, and the physical fatigue protocol consisted of five sets of smith-machine squats to failure using 70% of the participant’s body weight, with 60 s rest between sets. Mean peak scrum force decreased by about 13% due to physical fatigue or combined physical and mental fatigue, though mental fatigue alone did not significantly reduce scrum force. Green, Kerr, Olivier, et al. [27] used the Bath University Rugby Shuttle Test (BURST) as a game-simulation protocol to estimate the effects of fatigue due to game play on scrum force in individual university forwards. The BURST protocol requires players to do 5 min rounds of activity, including running, jogging, or walking and performing simulated contact situations such as mauls or tackles, with a very short rest period at the end of each round. In Green, Kerr, Olivier, et al.’s [27] study, university level players performed 16 cycles of the BURST protocol, divided into two halves with a 10 min rest period in between to simulate a real game. For data collection, participants performed two repetitions of 6 s maximal isometric scrummaging against a machine before and after the game-simulation protocol, with the trial for each testing period in which they produced the maximal force kept for analysis. Contrary to their hypothesis, they saw no significant reduction in scrummaging force after the game simulation, despite other markers of fatigue (blood lactate and rate of perceived exertion) significantly increasing. In a different study, Green, Dafkin, et al. [28] examined individual scrum force before and after players from local amateur clubs performed 10 live full-pack scrums, and also found no significant reduction in force despite two subjective indicators of fatigue (a visual analog scale of fatigue and ratings of perceived exertion scores) significantly increasing (*p* < 0.001) from the start to the end of the experiment.

## Discussion

The number of investigations into rugby scrums has increased dramatically since the last published literature review on the topic, which only included six studies that reported force production [7]. This interest reflects the international concern about safety during scrums, rise in interest and popularity of the sport, and a desire to understand effective performance during the scrum. With reintroduction of rugby 7 s into the Olympics, as well as the recent professionalization in the USA and the continued push for professionalization of women’s leagues in traditional rugby countries like England, the need for research-based evidence about safety and performance in the sport will continue to grow. There are a few major and general gaps in the current literature that will need be addressed to fill these needs: first, that most of the studies are done in elite male players and may not represent other populations (such as elite or amateur women); second, that many of the seminal studies were conducted prior to the law change about scrum engagement, and therefore may not represent force production under the modern call sequence of “crouch, bind, set” and subsequent coaching techniques and referee criteria; and third, that there is evidence that rugby players continue to get bigger and more physically developed than players who came before [37], which may limit the application of older studies’ results to the modern game.

To date, there has been a lack of standardization of procedures for testing scrum force. This is largely due to the fact that most research groups have had to custom-make their testing equipment, as there are very few commercially available instrumented scrum machines, and the only ones the authors are aware of are for individual scrummaging only and may not provide all the kinetics results researchers may be interested in. Live scrummaging has been especially difficult to capture. Future international collaboration and consensus is needed to help set standards to increase comparison and generalization among research studies. Despite the inherent face validity of testing force on an instrumented scrum machine, no study was found that examined the validity or sensitivity of measuring scrum force; only one study reported inter-trial reliability, and none reported inter-session reliability. Of the studies that did do multiple trials, and where change between individual trials was not relevant (e.g. not evaluating effect of fatigue), there was inconsistency in how repeated trials were treated; some studies used the best trial for later analysis [9, 14, 19, 26, 30], while others averaged across trials [10, 16, 20, 22, 23], and others failed to indicate or were not clear about how the repeated trials were treated [5, 6, 17, 21, 24, 31]. Furthermore, the conflicting results about whether repeated scrums cause fatigue brings into question the reliability of these tests. These properties should be determined.

Only a few studies investigated joint angles during the scrum [15, 16, 30, 35], and while there was a wide range, all of them reported knee and hip angles greater than 100°, with knee angles specifically ranging from 100.7–145° and hip angles ranging from 117.6–176°. These large angles for a task of high force production are similar to what other researchers have reported during resistance training tasks such as isometric squatting, where hip and knee angles of 120° or greater have demonstrated the greatest force production capability [39]. Having these larger joint angles likely provides the agonist muscles with a mechanical advantage and more optimal length-tension relationship, resulting in a player able to exert greater amounts of force compared to a player who starts in a position with smaller joint angles that may be more appropriate to more explosive activities like sprinting. Due to the minimization of the engagement phase in the scrum with recent rule changes, having the extra pre-scrum tension and taking advantage of the stretch-shortening cycle is likely no longer very beneficial. Instead, getting a longer body position more optimized for concentric pushing forward and for eccentric resistance to being driven back seems to be a better strategy. In the two studies that measured body height as well as joint angles during a scrum, the most advantageous knee and hip angles were achieved around 40% of body height [15, 35], with a wide stance. While this might represent an optimal position for an individual, or for a forward pack with little variation in player height, further research will be needed to investigate the interactions between pack members of different heights, where players may not be able to achieve the optimal individual height while successfully binding with their teammates (e.g. a tall lock trying to bind on to a much shorter prop). Knowledge of the combined positioning of pack members may help inform selection decisions of head coaches.

As expected, the summarized results showed that force production in the scrum increased with playing level. Discrepancies against this general trend are most likely due to variation in test conditions such as shoe type (running shoes vs. cleats vs. barefoot), ground surface (grass vs. artificial turf vs. tile floor vs. sprint blocks) and scrum scenario (live scrum with force measured with pressure sensors vs. machine scrum with instrumented pads). In studies that specifically compared scrum forces at different playing levels, this general trend of increasing scrum force with playing level was present [6, 11, 17, 30, 31, 40]. The increase in scrum force with increased playing level may be due to individual factors such as better player technique (and therefore greater ability to efficiently transmit ground reaction forces through the kinetic chain and apply it through the shoulders and arms), better rate coding and synchronization of high-threshold motor units, better intermuscular coordination [41], a higher ratio of lean body mass to total body mass, and greater overall body mass of individual players [18, 37], as well as synergy of individual players within the scrum pack. Argus and colleagues [18] found moderate differences between academy and professional players for lower body strength, and trivial to small differences between semi-professional and professional players for lower body strength and power, concluding that the majority of physical development (basic strength and power) can be achieved at the academy level after 1–2 years of structured training. Additionally, they found only small to moderate differences in mass between players of academy, semi-professional, and professional grade, and these differences in mass accounted for many of the differences in strength and power, as seen when they allometrically scaled their results for body mass. Assuming their samples are representative of strength, power, and body mass at these playing levels, the small observed differences in these characteristics likely do not fully explain the gap between these playing levels in maximal sustained force during machine scrummaging (Fig. 2e and f). Therefore, pack synergy, stability, and technique may be more important factors in scrum performance than individual force production capabilities for differentiating between these playing levels. Future research should identify valid and reliable methods for measuring individual scrum technique and, more importantly, total pack synergy in order to help distinguish factors that contribute to scrum performance.

This review can provide some normative values for coaches and players to compare their performance against. However, some changes in how study results are reported in the future would improve the utility of the research and help coaches apply the research to their practice. When reporting force, we recommend reporting absolute force in Newtons as well as force scaled for body mass in some respect, even if the optimal scaling method is yet to be determined for rugby [42, 43] or for the scrum. Scaled forces are likely to be useful for evaluating and comparing individual rugby players, even if it is the absolute forces in the scrum that ultimately determine pack motion. Comparisons between players of different pack positions (who may potentially have large differences in mass) may be more easily done with the inclusion of scaled force characteristics. Additionally, training decisions based on scrum data might be easier to make with the added context of scaled force data; for example, a decision for whether a player should get stronger without changing body mass or get stronger while also increasing body mass might be made easier if the coach knows how strong the player is relative to their mass. In situations where coaches or researchers only have the capability of testing players’ scrum force production individually, but want to estimate total pack scrum force production, it seems that a reasonable estimation can be gained by summing the individual forces when they are weighted. Based on the currently available evidence, weighting all players by 65% seems to be as good as weighting players specifically by factors related to their position for deriving a reasonable estimate. For research, to enable comparisons between studies, we strongly recommend not summing individuals’ forces as a percent of body mass (as was done in the study by Green, Dafkin et al. [28]).

The estimate of players contributing on average 65% of their individual force to the pack is primarily based on studies conducted 20 or more years ago [16, 31], and may not represent the modern player (who is generally more physically developed [37]) scrummaging under the modern engagement process. It would be worth replicating these seminal studies both in modern elite players and other populations as well, such as amateur women, to better understand current abilities and development needs of different rugby players. Furthermore, all the research so far has been conducted in samples from Rugby Union 15 s, and may not represent the demands of other codes of rugby like League or especially the three-man scrum used in 7 s; rugby 7 s likely has a unique set of demands on the players involved in the scrum and should become a priority due to the inclusion of rugby 7 s in the Olympics.

While this descriptive data can be used for comparisons and tracking player development, what is unfortunately missing from the literature is a robust exploration of the factors that contribute to on-field scrum performance. So far, the only study to examine the relationship between scrum force production and live scrum pack success measured the force production of individuals against an instrumented scrum machine, and determined scrum success as pushing the opposing back backwards but did not include putting in the ball [28]. Therefore, there is still a gap in the literature as to what factors actually contribute to winning possession in the scrum. Using individual scrum trials is potentially problematic, given that, the literature reviewed here indicates that summed individual forces, when not weighted by position or another method, do not accurately represent pack force production [14, 16, 31]. While the magnitude of difference in force production during a live scrum required by a pack in order to drive back the opposing pack remains unknown, from the literature reviewed here, it seems that a 10% or greater difference in force will lead to a shift in total scrum position of at least 1.5 m, potentially increasing the likelihood of gaining or maintaining possession of the ball. Future research will need to test to see if whole-pack force generation predicts scrum success, and what threshold difference between whole-pack force generation is needed to drive the opponent back and/or win the scrum.

Comparing the results of individual studies that tested either machine or live scrummaging show that during live scrums, teams produce about 50% of the force they could against a machine (see Fig. 2c vs a). The closest to a direct comparison of live and machine scrums comes from the pair of articles by Du Toit and colleagues [9, 14] that appear to have used the same sample under the two different conditions; they found that high school boys produced around 70% of their mean and max forces against a scrum machine when scrummaging live. This makes sense due to the stable and immovable nature of the machine, which facilitates maximal force production. In other strength testing settings, having a more stable surface also allowed for greater force production [44]. Furthermore, especially when testing individual players, pushing against a scrum machine likely does not reflect live scrum performance: against a machine individual players get to make contact with both shoulders and get into an optimal position for pushing; in a live scrum, players such as the flankers and the tight head prop only get to bind with one shoulder, and players may have to sacrifice individual ideal body position in order to fit with the other members of the pack. The surprising finding of this comparison was that professional players lost just as much proportional force production as amateur players (all playing levels produced about 50% of force during live compared to machines). One might expect that a professional pack as a whole would be more stable and synchronized, and therefore able to transfer force more effectively in a live scrum than an amateur pack. However, none of these findings can be taken conclusively because this review is primarily comparing data from separate studies. Thus, future research should test the same packs for both machine and live scrummaging to see what the relationship is between the two situations, so that coaches may know how well machine scrummaging may predict success in scrums in the live game.

Surprisingly, few studies have investigated individual factors that may contribute to scrum performance. In an absolute sense, it has been clearly documented that player, unit, and whole pack mass has a significant contribution to force generation in the scrum [9, 14–16], likely due to the greater inertia and increased force production benefit of the increased mass. However, a heavier pack is not guaranteed to win the scrum [28]. Only a few studies have examined how other physiological parameters, such as lower body power, may contribute to scrum force production [15, 16, 28]. Two out of three studies showed no correlation between vertical jump height and scrum force [15, 16], though combined vertical jump height of pack members does seem to be indicative of their ability to drive the opposing pack back [28]. One explanation for the lack of significant correlations between vertical jump and scrum force may simply be the mass of the players involved; large forwards may have so much body mass that they cannot jump high, even though they can produce a lot of force in a closed-kinetic chain situation (such as the scrum or traditional resistance training exercises like the squat). However, vertical jump height was still not correlated with sustained phase forces (r = 0.072) even when normalized to body mass and height [15]. A similar lack of correlation between jump height and other measures of strength and power has been seen in other strength athletes, such as wrestlers [45]. With both rugby players and wrestlers, one of the issues could be which positions/weight classes are specifically sampled from when performing the correlation analysis. For example, props are typically significantly heavier than flankers, so if players from both of those positions are included in the correlation, the variability in vertical jump height between those positions may indicate a non-significant correlation with scrum force. In contrast, if a sample consisted of only props or only flankers, a significant correlation between vertical jump and scrum force may be found due to reducing the standard error of the mean of vertical jump scores within the sample. Another factor that could explain the relatively consistent finding that vertical jumps do not correlate well with scrum performance could be the difference in external loading for both tasks and the substantially different place each lies along the force-velocity spectrum [46]. In other words, there may not be enough biomechanical specificity (e.g. similarity in velocity of task) between scrummaging and vertical jumps for the two tasks to demonstrate predictive validity. In support of this idea, Quarrie and Wilson [16] found that isokinetic knee extensions at 1.05 rads•s^− 1^ and 3.14 rads•s^− 1^ correlated more strongly to individual scrum performance (r = 0.39, 0.41) than did vertical jumps (r = − 0.13), in which knee angular velocities far exceed those rates [47]. One factor that has not been reported in the literature, which may help elucidate these distinctions further, is player body composition. While fat mass contributes to the inertia of a scrum pack, lean mass, of which skeletal muscle makes up a substantial proportion, is responsible for generating the force produced by each player and may be a better predictor of scrum force production than total body mass. Comparing force production capability based on lean mass may provide additional insight into the determinants of scrum performance, particularly when considering the influence of gender/sex. Recently, Nimphius [48] highlighted the importance of controlling for factors like training history, strength, and other modifiable characteristics when attempting to better understand the influence sex may have on performance. Given the paucity of scrum research on female rugby players, these additional fitness factors must be considered to gain a thorough and accurate understanding of scrum performance. Additionally, the relationship of other factors like cardiorespiratory fitness, rate of force development, and technique to scrum performance, especially to maintenance of scrum performance across an entire game, are worth investigating.

Combined results of studies examining the impact of fatigue on scrummaging have been equivocal so far. Four studies found a significant decrease in force due to fatigue, either from repeated scrums or some other fatigue protocol [21, 23, 29, 32]. However, when Morel conducted a follow up study, they found that they could eliminate decrements in scrum force production by simply providing at least 24 s of rest [33]. Other studies using various fatigue protocols have also shown no effect of fatigue on scrum force [27, 28]. Research examining the effect of game fatigue on other areas of physical and technical performance (such as tackling and ball handling) have sometimes found significant decrements over the course of the match [49], and other times only found trivial differences between first and second half performance [50]. While the studies on scrum fatigue represent a range of playing levels, most have been in elite players with a high level of physiological fitness and resistance to errors due to fatigue; therefore their results are likely not generalizable to other populations, especially those with less experience or lower fitness (e.g. American collegiate women brand new to the game). As a player fatigues, it is likely that they will contribute less pushing force in the scrum, but more importantly they may not be able to generate enough muscular stability to keep their spine in a safe alignment [22]. Physical performance characteristics such as maximum strength are known to play a role in a player’s resistance to fatigue and maintenance of rugby skills [51, 52]. Therefore, future research should examine the effect of fatigue not only on force production, but also body position and technique while scrummaging, while also considering the moderating effects of fitness characteristics like maximum strength on these changes. Additionally, all the current studies examining fatigue have measured players individually; there is a gap in looking at fatigue of the whole pack scrumming together as a unit. Furthermore, scrum outcomes during actual match play, instead of just a match-simulation protocol, should be examined to see how they change as the game progresses. The current evidence may indicate that referees should consider providing > 24 s rest between scrums that end in collapse or have some other need to be reset, especially when the match is being conducted among populations with expected lower levels of fitness.

The strengths of the present review include wide search criteria, not limiting results to English-language texts, the inclusion of gray literature, and the independent review of all studies by both authors. The limitations of the review include the search being executed by a single individual without confirmation by another, and having to conglomerate results from different though similar playing levels (e.g. pooling academy and semi-professional together) to have large enough groupings for generalized comparisons between the playing levels.

## Conclusion

To maximize force production during the sustained pushing phase of the scrum, players should seek to adopt a parallel foot position and achieve hip and knee angles (angle between the torso and thigh and angle between the thigh and leg, respectively, as would be measured by a goniometer) each of approximately 120°. These angles will likely be achieved when setting the shoulders at a height of 40% of player body height, though proper binding with the other pack members should take precedence over achieving this particular position. To minimize fatigue that may occur when successive scrums are required due to ball straight out, collapsed or wheeled scrums, etc., we recommend that referees provide at least 24 s rest between the scrums to minimize any risk of injury that may be created by multiple scrums. For testing protocols, we recommend when possible testing rugby players on whatever surface they are most likely to play on, wearing their cleats, and without foot support to maximally replicate game-day conditions. For future research in this area, we recommend focussing on full pack scrummaging, discovering what factors are most important to scrum success, and on designing studies so that laboratory measures are paired with live-scrum situations to determine the influence on the real game. A paucity of scrum research on female rugby players has necessitated that researchers and practitioners operate on the assumption that scrum research on males can be generalized to females; this is a potentially fallacious assumption to make, and significant further research is needed on female rugby players. Finally, scrum success has so far been measured as pushing an opponent backward, but in the future needs to include the throw in and contest for possession.

## Data Availability

All data generated or analysed during this study are included in this published article in Table 1.

## Abbreviations

BURST: Bath University Rugby Shuttle Test
NIH: National Institutes of Health

## References

[1] World Rugby (2018). Laws of the game Rugby union.

[2] Rugby League International Federation (2017). International laws of the game with notes on the laws.

[3] Ortega E, Villarejo D, Palao JM (2009). Differences in game statistics between winning and losing rugby teams in the six nations tournament. J Sports Sci Med.

[4] Coughlan M, Mountifield C, Sharpe S, Mara JK (2019). How they scored the tries: applying cluster analysis to identify playing patterns that lead to tries in super rugby. Int J Perform Anal Sport.

[5] Cazzola D, Preatoni E, Stokes KA, England ME, Trewartha G (2015). A modified prebind engagement process reduces biomechanical loading on front row players during scrummaging: a cross-sectional study of 11 elite teams. Br J Sports Med.

[6] Preatoni E, Stokes KA, England ME, Trewartha G (2015). Engagement techniques and playing level impact the biomechanical demands on rugby forwards during machine-based scrummaging. Br J Sports Med.

[7] Trewartha G, Preatoni E, England ME, Stokes KA (2015). Injury and biomechanical perspectives on the rugby scrum: a review of the literature. Br J Sports Med.

[8] Hendricks S, Lambert MI, Brown JC, Readhead C, Viljoen W (2014). An evidence-driven approach to scrum law modifications in amateur rugby played in South Africa. Br J Sports Med.

[9] Du Toit DE, Olivier PE, Buys FJ (2005). Kinetics of full scrum and staggered scrum engagement in under 19 schoolboy rugby union players. S Afr J Res Sport Phys Ed Rec.

[10] Hodge KP (1981). Spinal injuries in rugby scrums. N Z J Health Phys Ed Rec.

[11] Preatoni E, Cazzola D, Stokes KA, England M, Trewartha G (2016). Pre-binding prior to full engagement improves loading conditions for front-row players in contested Rugby union scrums. Scand J Med Sci Sports.

[12] Bradley EJ, Hogg B, Archer DT (2018). Effect of the PreBind engagement process on scrum timing and stability in the 2013–16 six nations. Int J Sports Physiol Perform.

[13] Stean D, Barnes A, Churchill SM (2015). Effect of the “crouch, bind, set” engagement routine on scrum performance in English premiership Rugby. Int J Perform Anal Sport.

[14] Du Toit DE, Venter DJL, Buys FJ, Olivier PE (2004). Kinetics of rugby union scrumming in under 19 schoolboy rugby forwards. S Afr J Res Sport Phys Ed Rec.

[15] Green A, Kerr S, Dafkin C, Olivier B, McKinon W (2017). A lower body height and wider foot stance are positively associated with the generation of individual scrummaging forces in rugby. Int J Perform Anal Sport.

[16] Quarrie KL, Wilson BD (2000). Force production in the rugby union scrum. J Sports Sci.

[17] Preatoni E, Stokes KA, England ME, Trewartha G (2013). The influence of playing level on the biomechanical demands experienced by rugby union forwards during machine scrummaging. Scand J Med Sci Sports.

[18] Argus CK, Gill ND, Keogh JWL (2012). Characterization of the differences in strength and power between different levels of competition in rugby union athletes. J Strength Cond Res.

[19] Babault N, Cometti G, Bernardin M, Pousson M, Chatard J (2007). Effects of electromyostimulation training on muscle strength and power of elite rugby players. J Strength Cond Res.

[20] Bayne H, Kat CJ (2018). The influence of foot position on scrum kinetics during machine scrummaging. J Sports Sci.

[21] Birch CO (2016). How does mental and physical fatigue affect a rugby player’s force production during scrummaging? [masters].

[22] Clayton JD (2016). The influence of hip mobility and fatigue on spinal flexion and muscle activation in Rugby scrum performance [masters].

[23] Cochrane DJ, Harnett K, Lopez-Villalobos N, Hapeta J (2017). The effect of repetitive rugby scrummaging on force output and muscle activity. Sports Med Int Open.

[24] Dobbs I (2017). Correlation between isometric horizontal push force and sprinting across positions in collegiate rugby union players [masters].

[25] Ferrandino M, Forrester S, Fleming P (2015). The player surface interaction of rugby players with 3g artificial turf during rugby specific movements. Procedia Eng.

[26] Green Andrew, Kerr Samantha, Dafkin Chloe, McKinon Warrick (2015). The calibration and application of an individual scrummaging ergometer. Sports Engineering.

[27] Green A, Kerr S, Olivier B, Meiring R, Dafkin C, McKinon W (2017). A simulated rugby match protocol induces physiological fatigue without decreased individual scrummaging performance. S Afr J Sports Med.

[28] Green A, Dafkin C, Kerr S, McKinon W (2017). Combined individual scrummaging kinetics and muscular power predict competitive team scrum success. Eur J Sport Sci.

[29] Lacome M (2013). Analyse de la tâche et physiologie appliquée au rugby : étude de la fatigue associée à l’exercice maximal isométrique répété [Doctorate].

[30] Mensaert S, Sharp T, Vanwanseele B, Colloud F, Domalain M, Monnet T (2015). Influence of playing level on the kinematics and kinetics of the rugby scrum. Proceedings of the 33rd international conference on biomechanics in sports, 2015 June 29–July 3; Poitiers, France.

[31] Milburn PD (1990). The kinetics of rugby union scrummaging. J Sports Sci.

[32] Morel B, Rouffet DM, Bishop DJ, Rota SJ, Hautier CA (2015). Fatigue induced by repeated maximal efforts is specific to the rugby task performed. Int J Sports Sci Coach.

[33] Morel B, Hautier CA (2017). The neuromuscular fatigue induced by repeated scrums generates instability that can be limited by appropriate recovery. Scand J Med Sci Sports.

[34] Saletti D, Chicoulaa G, Raszoudowsky M, Drevelle X, Piscione J, Retière D (2013). Kinematic and dynamic responses of the scrum. Comput Methods Biomech Biomed Engin.

[35] Wu WL, Chang JJ, Wu JH, Guo LY (2007). An investigation of rugby scrummaging posture and individual maximum pushing force. J Strength Cond Res.

[36] Cazzola D, Trewartha G, Preatoni E (2014). Time-based calibrations of pressure sensors improve the estimation of force signals containing impulsive events. Proc Inst Mech Eng P J Sport Eng Technol.

[37] Smart DJ, Hopkins WG, Gill ND (2013). Differences and changes in the physical characteristics of professional and amateur rugby union players. J Strength Cond Res.

[38] Milburn PD (1993). Biomechanics of Rugby union scrummaging. Sports Med.

[39] Drake D, Kennedy R, Wallace E (2017). The validity and responsiveness of isometric lower body multi-joint tests of muscular strength: a systematic review. Sports Med Open.

[40] Sharp T, Halaki M, Greene A, Vanwanseele B (2014). An EMG assessment of front row Rugby union scrummaging. Int J Perform Anal Sport.

[41] Yaghoubi M, Lark SD, Page WH, Fink PW, Shultz SP (2019). Lower extremity muscle function of front row rugby union scrummaging. Sports Biomech.

[42] Comfort P, Pearson SJ (2014). Scaling- which methods best predict performance?. J Strength Cond Res.

[43] Crewther BT, Kilduff LP, Cook CJ, Cunningham DJ, Bunce PJ, Bracken RM (2012). Scaling strength and power for body mass differences in rugby union players. J Sports Med Phys Fitness.

[44] McBride JM, Cormie P, Deane R (2006). Isometric squat force output and muscle activity in stable and unstable conditions. J Strength Cond Res.

[45] Stockbrugger BA, Haennel RG (2003). Contributing factors to performance of a medicine ball explosive power test: a comparison between jump and nonjump athletes. J Strength Cond Res.

[46] Haff GG, Nimphius S (2012). Training principles for power. Strength Cond J.

[47] Rodacki AL, Fowler NE, Bennett SJ (2002). Vertical jump coordination: fatigue effects. Med Sci Sports Exerc.

[48] Nimphius S (2019). Exercise and sport science failing by design in understanding female athletes. Int J Sports Physiol Perform.

[49] Kempton T, Sirotic AC, Cameron M, Coutts AJ (2013). Match-related fatigue reduces physical and technical performance during elite rugby league match-play: a case study. J Sports Sci.

[50] Lacome M, Piscione J, Hager J-P, Carling C (2017). Fluctuations in running and skill-related performance in elite rugby union match-play. Eur J Sport Sci.

[51] Gabbett TJ (2016). Influence of fatigue on tackling ability in Rugby league players: role of muscular strength, endurance, and aerobic qualities. PLoS One.

[52] Gabbett TJ (2008). Influence of fatigue on tackling technique in rugby league players. J Strength Cond Res.

